# The Copper Amine Oxidase AtCuAOδ Participates in Abscisic Acid-Induced Stomatal Closure in Arabidopsis

**DOI:** 10.3390/plants8060183

**Published:** 2019-06-20

**Authors:** Ilaria Fraudentali, Sandip A. Ghuge, Andrea Carucci, Paraskevi Tavladoraki, Riccardo Angelini, Alessandra Cona, Renato A. Rodrigues-Pousada

**Affiliations:** 1 Department of Science, Università Roma Tre, 00146 Roma, Italy; ilaria.fraudentali@uniroma3.it (I.F.); andrea.carucci@outlook.it (A.C.); paraskevi.tavladoraki@uniroma3.it (P.T.); riccardo.angelini@uniroma3.it (R.A.); alessandra.cona@uniroma3.it (A.C.); 2 Institute of Plant Sciences, The Volcani Center, ARO, Bet Dagan 50250, Israel; sandip.ghuge.biotech@gmail.com; 3 Istituto Nazionale Biostrutture e Biosistemi (INBB), 00136 Rome, Italy; 4 Department of Life, Health, and Environmental Sciences, Università dell’Aquila, 67100 L’Aquila, Italy

**Keywords:** copper amine oxidases, H_2_O_2_, ROS, polyamines, ABA, stomatal closure

## Abstract

Plant copper amine oxidases (CuAOs) are involved in wound healing, defense against pathogens, methyl-jasmonate-induced protoxylem differentiation, and abscisic acid (ABA)-induced stomatal closure. In the present study, we investigated the role of the *Arabidopsis thaliana* CuAOδ (AtCuAOδ; At4g12290) in the ABA-mediated stomatal closure by genetic and pharmacological approaches. Obtained data show that *AtCuAOδ* is up-regulated by ABA and that two *Atcuaoδ* T-DNA insertional mutants are less responsive to this hormone, showing reduced ABA-mediated stomatal closure and H_2_O_2_ accumulation in guard cells as compared to the wild-type (WT) plants. Furthermore, CuAO inhibitors, as well as the hydrogen peroxide (H_2_O_2_) scavenger *N,N^1^*-dimethylthiourea, reversed most of the ABA-induced stomatal closure in WT plants. Consistently, *AtCuAOδ* over-expressing transgenic plants display a constitutively increased stomatal closure and increased H_2_O_2_ production compared to WT plants. Our data suggest that AtCuAOδ is involved in the H_2_O_2_ production related to ABA-induced stomatal closure.

## 1. Introduction

Copper amine oxidases (CuAOs) are dimeric proteins of 140–180 kDa, containing a copper ion and a redox-active organic cofactor 2,4,5-trihydroxyphenylalanine quinone (TPQ) for each monomer. These enzymes catalyze the intracellular and extracellular terminal catabolism of amines, including monoamines, diamines, and polyamines (PAs), by oxidizing the carbon next to the primary amino group, with the subsequent reduction of molecular oxygen to hydrogen peroxide (H_2_O_2_) and the production of the corresponding aldehydes and ammonia [1,2]. CuAOs have been found at high expression levels in several species of *Fabaceae*, especially in the cell wall of pea (*Pisum sativum*), chickpea (*Cicer arietinum*), lentil (*Lens culinaris*), and soybean (*Glycine max*) seedlings, from which these enzymes have been purified and characterized [3]. CuAOs from these species preferentially oxidize the diamine putrescine (Put) and cadaverine [4]. A number of peroxisomal and apoplastic CuAOs have been described in Arabidopsis (*Arabidopsis thaliana*) [5,6,7,8], tobacco (*Nicotiana tabacum*) [8,9], and apple (*Malus domestica*) [10], and a latex CuAO has been characterized from Mediterranean spurge (*Euphorbia characias*) [11], with diverse substrate affinities and specificities [12].

CuAOs belong to the larger family of amine oxidases (AOs), which also includes flavin adenine dinucleotide (FAD)-dependent polyamine oxidases (PAOs) [3]. The latter enzymes oxidize PAs to aminoaldehydes at the carbon neighboring the secondary amino group, resulting in the production of different residual amine moieties, depending on the location of the oxidized carbon in the aliphatic chain [1,2,12]. The other major co-product of the PAO-catalyzed PA oxidation reaction is hydrogen peroxide (H_2_O_2_) [2].

In spite of differences in biochemical features, such as protein structure, co-factors, and catalytic mechanisms, CuAOs and PAOs partially share substrates and reaction products and play overlapping roles in both the intracellular control of PA’s homeostasis and production of biologically active compounds and metabolites, such as the developmentally-controlled or stress-induced H_2_O_2_ [1,2,3]. Regarding this, the PA-derived apoplastic H_2_O_2_ has been proposed to act as both a signal for activation of defense gene expression and as a co-substrate for the peroxidase-driven reactions of wall-stiffening and lignification events [3]. In particular, plant AOs have been involved in a variety of growth and developmental events, including light-induced inhibition of mesocotyl growth [13], root xylem differentiation [14] and pollen tube growth [15], as well as in stress tolerance and defense responses, especially salt stress [16], wounding [17], pathogen attack [9,18,19,20,21], and stomatal closure [22,23,24,25].

Ten putative *CuAO* genes are annotated in the Arabidopsis genome, four of which have been characterized for substrate specificity and subcellular localization of the encoded enzymes and regulation of gene expression. The apoplastic AtCuAOβ (formerly AtAO1; At4g14940) [5] and AtCuAOγ1 (formerly AtCuAO1; At1g62810), the peroxisomal AtCuAOα3 (formerly AtCuAO2; At1g31710), and AtCuAOζ (formerly AtCuAO3; At2g42490) [7,12,25] all oxidize Spd at the primary amino group with an affinity comparable to that for Put. Expression of these AtCuAO-encoding genes is inducible by stress-related hormones and elicitors, such as methyl-jasmonate (MeJA; *AtCuAOβ*, *AtCuAOγ1*, *AtCuAOα3* and *AtCuAOζ*), abscisic acid (ABA), salicylic acid, and flagellin 22 (*AtCuAOγ1*, *AtCuAOζ*), and by wounding (*AtCuAOα3*) [7,26]. Notwithstanding the high number of annotated genes, only a few studies concerning the physiological roles of *AtCuAOs* have been reported so far. In this regard, it has been described that *AtCuAOγ1* and *AtCuAOζ* are involved in the ABA-mediated stress responses by contributing respectively to the ABA-induced production of nitric oxide (NO) [27] and the ABA-induced stomatal closure [25]. Furthermore, it has been shown that the AtCuAOβ-driven production of apoplastic H_2_O_2_ signals the MeJA-mediated protoxylem differentiation in Arabidopsis roots [26,28]. In this regard, *AtCuAOβ* gene expression in guard cells of leaves and flowers has been demonstrated, suggesting a role for this gene also in the control of stomatal closure [29]. Concerning the other *AtCuAO* annotated genes, the gene product of *AtCuAOδ* (At4g12290) has been identified among proteins purified from the central vacuoles of rosette leaf tissue by means of complementary proteomic methodologies [30].

The role played by the vacuole in ABA-induced stomatal closure [31], along with the occurrence of an ABA-inducible *AtCuAOδ* expression in guard cells, as reported by the Arabidopsis eFP Browser (http://bar.utoronto.ca/efp/cgi-bin/efpWeb.cgi; [32]), led us to analyze the possible involvement of the vacuolar AtCuAOδ in the control of stomatal movement. Herein we provide genetic and physiological evidence for a role of this protein as a H_2_O_2_ source in the ABA-induced stomatal closure.

## 2. Results

### 2.1. AtCuAOδ Expression Is Induced by ABA

**Figure 1:**
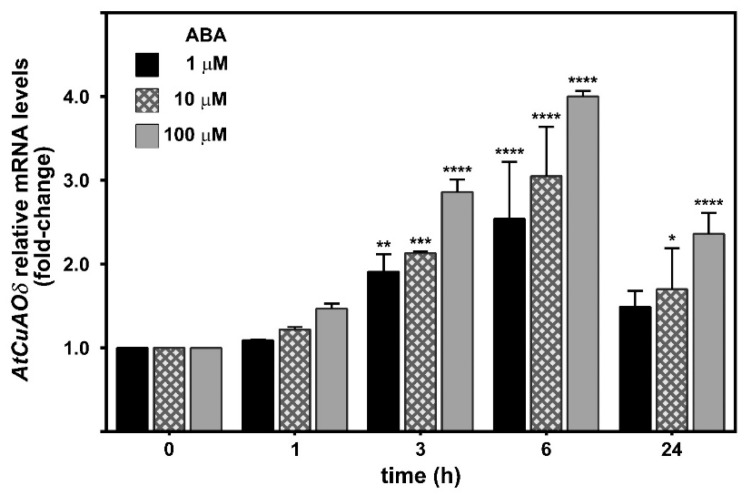
Analysis of *AtCuAOδ* gene expression upon abscisic acid (ABA) treatment by reverse transcription-quantitative polymerase chain reaction (RT-qPCR). The expression of *AtCuAOδ* gene was analyzed in 12-day-old wild-type (WT) seedlings untreated or treated with 1, 10, and 100 µM ABA for 0, 1, 3, 6, and 24 h. Five independent experiments as biological replicates (mean values ± SD; *n* = 5) were performed. *AtCuAOδ* mRNA level after ABA treatment is relative to that of the corresponding untreated plant for each time point. The significance levels between the relative mRNA level at each time and the mRNA level of control untreated plant at time 0, which is assumed to be one, is reported. *P* values have been calculated with one-way analysis of variance (ANOVA); *, **, ***, and **** *p* values equal or are less than 0.05, 0.01, 0.001, and 0.0001, respectively.

A promoter region of approximately 2.7 kb upstream of the *AtCuAOδ* start codon was analyzed in silico for the presence of cis-acting elements by the Arabidopsis eFP Browser (http://bar.utoronto.ca/cistome/cgi-bin/BAR_Cistome.cgi). On the basis of this analysis, two recognition sequences (CATGTG) for the ABA-inducible MYC factor (MYCATERD1) necessary for the expression of *erd1* (early responsive to dehydration) in dehydrated Arabidopsis plants were identified. Moreover, the analysis of microarray data retrieved from the Arabidopsis eFP Browser revealed the occurrence of *AtCuAOδ* mRNA in guard cells, whose level increased upon ABA-treatment. These data are supported by reverse transcription-quantitative polymerase chain reaction (RT-qPCR) studies that showed a two- to three-fold increase of *AtCuAOδ* expression levels depending on ABA concentration as soon as 3 h after the onset of treatment (Figure 1). This induction peaked at 6 h with a four-fold increase at 100 µM ABA, and returned to almost control levels at 24 h for the two lower concentrations while it was still two-fold higher at 100 µM ABA.

### 2.2. AtCuAOδ Loss-of-Function Mutants Are Unresponsive to ABA-Induced Stomatal Closure 

In order to investigate the contribution of *AtCuAOδ* in ABA-mediated responses, two T-DNA insertional mutant lines for this gene [SALK 072954.55.00.x line, TAIR (The Arabidopsis Information Resource) accession number 4122972 and GK-011C04-013046 line, TAIR accession number 4242275] were identified from the TAIR database (http://www.arabidopsis.org/; [33]), and obtained, hereafter referred to as *Atcuaoδ.1* and *Atcuaoδ.2* (Figure S1). From evidence available in TAIR, the T-DNA insertion sites are located in the first exon in both mutants (Figure S1), upstream of the encoded catalytic site active residues (Figure S2), thereby creating loss-of-function mutants. Mutant plants homozygous for the T-DNA insertions were identified by PCR analysis of genomic DNA (Figure S1). RT-PCR analysis of the selected plants confirmed the absence of the full-length gene transcripts in *Atcuaoδ.1* and *Atcuaoδ.2* (Figure S1). Analysis of *Atcuaoδ* mutants under physiological growth conditions did not highlight any apparent different phenotypes, i.e., germination events, stem or root length, and leaf morphology (data not shown).

**Figure 2:**
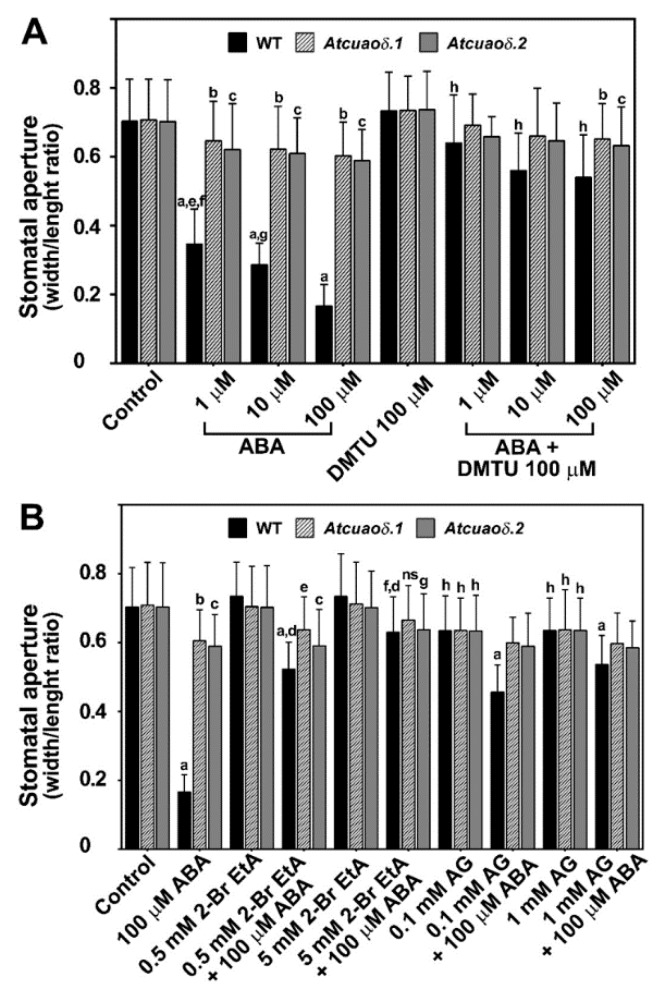
Effect of ABA, *N,N*′-dimethylthiourea (DMTU) (**A**) and CuAO inhibitors, 2-BrEtA and aminoguanidine (AG) (**B**), on stomatal pore width/length ratio of 12-day-old seedlings from WT, *Atcuaoδ.1*, and *Atcuaoδ.2.* Mean values ± SD (*n* = 15) are reported. *P* values have been calculated with one-way ANOVA analysis; non-significant (ns) differences: *p* values > 0.05; *, **, ***, and **** *p* values are equal to or less than 0.05, 0.01, 0.001, and 0.0001, respectively. (**A**) Seedlings were treated for 2 h with ABA (1, 10, and 100 µM) and DMTU (100 µM), either alone or in combination with the hormone. The significance levels are described with letters where appropriate; non-significant differences are not indicated; a = ****, ABA 1, 10, and 100 µM WT vs. Control WT; b = **, ABA 1 µM/ABA 100 µM + DMTU *Atcuaoδ.1* vs. Control *Atcuaoδ.1*; c = ****, ABA 1, 10, 100 µM *Atcuaoδ.2*/ABA 100 µM + DMTU *Atcuaoδ.2* vs. Control *Atcuaoδ.2*; d= ****, ABA 10, 100 µM *Atcuaoδ.1* vs. Control *Atcuaoδ.1*; e = *, ABA 1 µM WT vs. ABA 10 µM WT; f = ****, ABA 1 µM WT vs. ABA 100 µM WT; g = ****, ABA 10 µM WT vs. ABA 100 µM WT; h = ****, ABA 1, 10, and 100 µM WT vs. ABA 1, 10, and 100 µM + DMTU WT. (**B**) Seedlings were treated with 2-BrEtA (0.5, 5 mM) or AG (0.1, 1 mM) for 30 min. ABA was added (100 μM) and further incubated for 2 h. The significance levels are described with letters where appropriate.; ns: ABA 100 µM + 5 mM 2-BrEtA *Atcuaoδ.1* vs. Control *Atcuaoδ.1*; a: ****, ABA 100 µM, ABA 100 µM + 0.5 mM 2-BrEtA, ABA 100 µM + AG 0.1 or 1 mM WT vs. Control WT; b: ****, ABA 100 µM *Atcuaoδ.1* vs. Control *Atcuaoδ.1*; c: ****, ABA 100 µM/ABA 100 µM + 0.5mM 2-BrEtA *Atcuaoδ.2* vs. Control *Atcuaoδ.2*; d: ****, ABA 100 µM + 2-BrEtA 0.5 or 5 mM WT vs. ABA 100 µM WT; e: ** ABA 100 µM + 0.5 mM 2-BrEtA *Atcuaoδ.1* vs. Control *Atcuaoδ.1*; f: ** ABA 100 µM + 2-BrEtA 5 mM WT vs. Control WT; g: * ABA 100 µM + 2-BrEtA 5 mM *Atcuaoδ.2* vs. Control *Atcuaoδ.2*; h: *, AG 0.1 or 1 mM, WT, *Atcuaoδ.1* e *Atcuaoδ.2* vs. Control WT, Control *Atcuaoδ.1*, or Control *Atcuaoδ.2.*

As ABA has well characterized effects in the regulation of the stomatal aperture, we investigated whether AtCuAOδ-driven PA oxidation in guard cells could be involved in the ABA-mediated stomatal closure. Wild-type (WT), *Atcuaoδ.1*, and *Atcuaoδ.2* plants were treated with ABA (1, 10, and 100 µM) for 2 h and the stomatal aperture was analyzed by measuring the width and the length of the stomatal pore (width/length ratio). In Figure 2A and Table S1, we show that while between control untreated WT (Control WT) and control untreated insertional mutants (Control *Atcuaoδ.1* or Control *Atcuaoδ.2)* no width/length ratio differences were detected, a significant reduction of the ABA-mediated stomatal closure was observed in the *Atcuaoδ* mutants as compared to WT plants. Indeed, stomatal closure was induced in WT by ABA treatments of about 51% at 1 µM and 77% at 100 µM as compared to Control WT, while the ABA-mediated stomatal closure in *Atcuaoδ.1* and in *Atcuaoδ.2* ranged from ~9–12% to a maximum of ~15–17%, respectively, as compared to the corresponding Control mutant plants (Figure 2A).

Consistent with these data, treatment with the CuAO-specific inhibitors, 2-bromoethylamine (2-BrEtA) and aminoguanidine (AG), inhibited the ABA-mediated stomatal closure in WT plants (Figure 2B and Table S2). The action of the CuAO-specific inhibitors, 2-BrEtA and AG, on stomatal apertures of *Atcuaoδ* mutants was also studied. At the two different inhibitor concentrations used, in the presence of 100 µM ABA (the highest hormone concentration used), we observed diverse antagonistic effects. At the 2-BrEtA highest concentration, a considerable reduction of stomatal closure was observed in WT (from 77% to 10%), while the mutant genotypes were further unresponsive to ABA (5% from 15% in *Atcuaoδ.1* and 9% from 17% in *Atcuaoδ.2*). The other CuAO inhibitor, AG, partially prevented the stomatal closure effects induced by ABA in WT (35% and 24% at 0.1 mM and 1 mM, respectively). However, by itself this inhibitor presented a similar effect on stomatal apertures in all the studied genotypes (10% closure in respect to the Control WT and mutant).

### 2.3. AtCuAOδ-Driven Production of H_2_O_2_ Is Involved in the ABA-Induced Stomatal Closure

**Figure 3:**
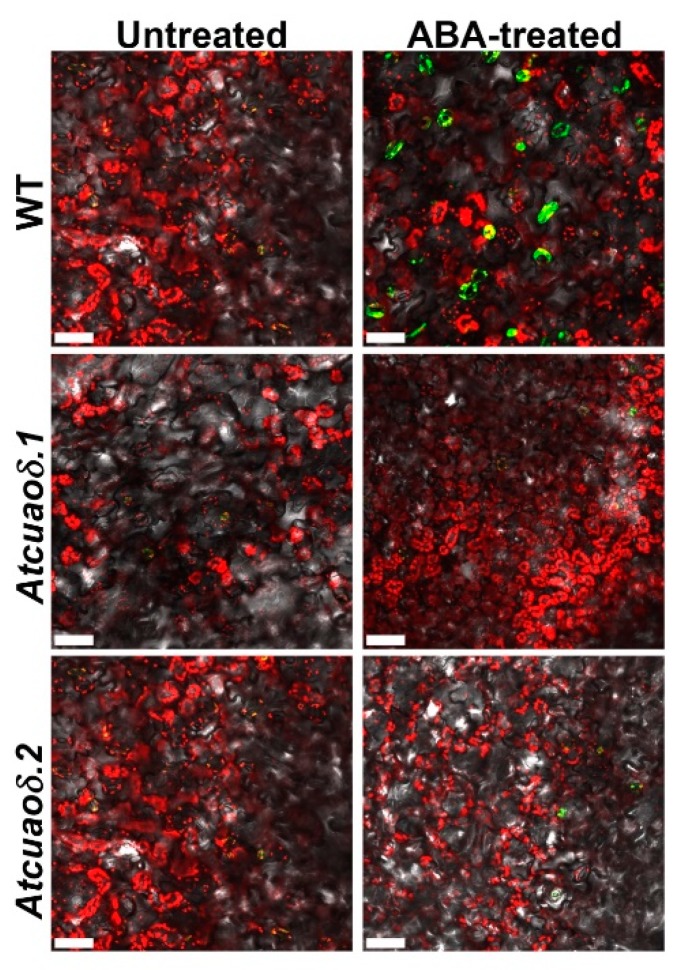
Reactive oxygen species (ROS) levels in guard cells of leaves from 12-day-old seedling. In situ ROS detection in guard cells by Laser Scanning Confocal Microscopy (LSCM) analysis after 2′,7′-dichlorodihydrofluorescein diacetate (CM-H_2_DCFDA) staining of leaves from WT, *Atcuaoδ.1*, and *Atcuaoδ.2*, untreated, or 100 µM ABA-treated plants (2 h; green-yellow). Micrographs are representative of those obtained from five independent experiments, each time analyzing leaves from five plants per genotype and treatment. Bar = 50 µm.

In order to get insights into the possible role played by the AtCuAOδ-driven production of H_2_O_2_ in the stomatal closure induced by ABA, WT and *Atcuaoδ* seedlings were treated with the H_2_O_2_ scavenger *N,N^1^*-dimethylthiourea (DMTU) at the working concentration of 100 µM [26], either alone or in combination with 1, 10, and 100 µM ABA. DMTU reversed the ABA-induced stomatal closure in WT plants (91, 80, and 78%, respectively), whereas it did not significantly affect stomatal aperture under physiological conditions in WT plants, or in 1 and 10 µM ABA-treated and untreated mutants (Figure 2A and Table S1). At the highest ABA concentration used, DMTU reversion was not complete in the mutant genotypes, where a significant closure effect of 8% and 11% was observed in *Atcuaoδ.1* and *Atcuaoδ.2*, respectively. To further investigate the contribution of AtCuAOδ in the ABA-induced H_2_O_2_ production, reactive oxygen species (ROS) levels in guard cells were analyzed using a chloromethyl derivative of 2′,7′-dichlorodihydrofluorescein diacetate (CM-H_2_DCFDA). Figure 3 shows ROS levels through Laser Scanning Confocal Microscopy (LSCM) analysis in both WT and *Atcuaoδ* mutant plants. Under the technical conditions of our analysis, ROS were undetectable in Control WT and Control mutants (Figure 3 left panels), while 100 µM ABA-treatment induced ROS in guard cells of WT plants (Figure 3, upper right panel), as indicated by the green-yellow colored stomata. The signal was absent in *Atcuaoδ.1 and Atcuaoδ.2* mutant seedlings (Figure 3, right lower panels).

### 2.4. AtCuAOδ Over-Expressing Plants Show Enhanced Stomatal Closure and H_2_O_2_ Production

**Figure 4:**
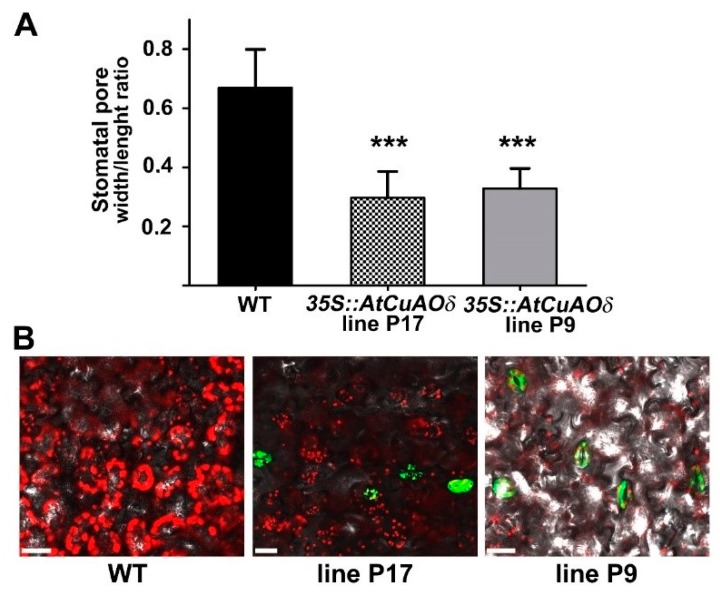
Effect of *AtCuAOδ* over-expression on stomatal pore width/length ratio and ROS levels in guard cells of leaves from 12-day-old WT and *overAtCuAOδ* seedlings. (**A**) Leaves from WT and *overAtCuAOδ* lines P17 and P9 were incubated in the opening solution for 3 h under light to allow stomata opening and then incubated with the fixing solution. Then, the width/length stomatal ratio was measured. Mean values ± SD (*n* = 15) are reported. The significance levels between WT and *overAtCuAOδ* plants are reported. *P* values have been calculated with one-way ANOVA analysis; ***, *p* value equal or less than 0.001. (**B**) In situ ROS detection in guard cells by LSCM analysis after CM-H_2_DCFDA staining (green-yellow) of leaves from WT and *overAtCuAOδ* lines (P17 and P9). Micrographs are representative of those obtained from five independent experiments, each time analyzing leaves from five plants per genotype and treatment. Bar = 25 µm.

The role played by AtCuAOδ-driven production of H_2_O_2_ in the stomatal closure has been further investigated through the analysis of transgenic Arabidopsis plants over-expressing *AtCuAOδ* (*overAtCuAOδ*). In Figure S3 we show by RT-qPCR and western blot analysis the different levels of the transgene mRNA and protein of the two lines, *overAtCuAOδ* line P9 and *overAtCuAOδ* line P17, used in this work. The latter showed higher expression of the transgenic mRNA and protein than *overAtCuAOδ* line P9 (Figure S3). As shown in Figure 4, both *overAtCuAOδ* lines showed constitutively enhanced stomatal closure (Figure 4A) and similar ROS production in guard cells (Figure 4B) as compared to WT plants.

## 3. Discussion

### 3.1. AtCuAOδ Plays a Role in the Control of Stomatal Closure in Response to ABA

Our data (Figure 1) show that *AtCuAOδ* gene is regulated by ABA, consistent with the ABA-regulated recognition sites identified in its promoter region. In detail, a maximum of 2.5- to four-fold induction, depending on the ABA concentration used, was observed after 6 h from the onset of treatment. In this regard, it is known that ABA, the water-stress hormonal signal that is considered a valid indicator of water potential status in plants, is involved in defense responses against abiotic stresses, such as drought or high soil saline levels [34,35,36]. In line with this, several results have suggested that the ABA-responsive AOs [22,25] are likely involved in salt stress responses [37] and in water balance regulation [28].

Furthermore, one of the major roles of this phytohormone is its action on the regulation of stomatal movement in response to variations in water potential [38,39]. Regarding this aspect of plant responses to ABA, our results demonstrate that alterations in the levels of *AtCuAOδ* expression by reverse genetics and over-expression approaches, or AtCuAO enzyme activities by pharmacological treatments with the two known CuAO activity inhibitors, 2-BrEtA or AG [40,41,42], caused alterations of the hormonal control of guard cells responses. In fact, it was observed a complete to partial unresponsiveness to ABA in stomatal closure with the two homozygous *Atcuaoδ* mutants, compared to the WT (Figure 2A and Table S1), and the lack of ABA responsiveness in the WT in combination treatments involving the two inhibitors (Figure 2B and Table S2). It must be pointed out that this effect was clear even in the case of AG, even if this inhibitor caused by itself a similar stomatal closure effect on all the three studied genotypes (approximately 10% of Control WT and Control mutants). This unspecific effect could be attributed to AG-induced alterations of the plasma membrane potential [43], which could influence stomatal movements. The *overAtCuAOδ* lines instead showed a significantly reduced (50%) stomatal aperture in respect to WT (Figure 4A).

### 3.2. Vacuolar AtCuAOδ-Dependent H_2_O_2_ Production Is a Necessary Condition for ABA Regulation of Stomatal Aperture

Figure 3 and Figure 4 show that when compared to the WT, the loss-of-function or the over-expression of the gene encoding the vacuole-resident AtCuAOδ [29] reduced ROS levels of guard cells in ABA-treated mutant genotypes or increased ROS in *overAtCuAOδ* lines. ROS, such as H_2_O_2_, are ubiquitous metabolites in all aerobic organisms and have been shown to be important signals in many aspects of plant development, including the regulation of stomatal movement [44,45,46]. Thus, the relation between AtCuAOδ-mediated H_2_O_2_ production and its involvement in stomatal closure in the ABA transduction pathway is consistent with the role of H_2_O_2_ in ABA-signaled phenomena.

Interestingly, vacuoles have an important role in the regulation of stomatal pore apertures associated with different environmental or hormonal factors signaling water stress [47,48,49]. Moreover, ROS can regulate several channel activities located in the tonoplast, which influence ion fluxes, cytosolic pH, and the uptake and release of calcium [30], all of which are involved in modulation of the stomatal aperture. It is, thus, not surprising that an AtCuAO protein identified in the vacuolar proteome [29] can influence stomatal closure as an element in the ABA transduction pathway regulating this phenomenon. No clear contribution of vacuoles in guard cells to the ROS signaling network has been identified [46]. Nevertheless, reports in the literature indicate that vacuoles can be sites of H_2_O_2_ production [50,51,52]. Thus, our data might represent a first indication that ROS in the form of H_2_O_2_ produced by a vacuolar AtCuAO have a physiological role in ABA regulation of stomatal movement.

### 3.3. Vacuolar AtCuAOδ Cooperates with Different ROS Sources in Regulation of Stomatal Movement

Among CuAO family member AtCuAOδ is not the only CuAO involved in the control of stomatal aperture levels. Indeed, both *AtCuAOβ* and *AtCuAOζ* are expressed in guard cells [25,29], and in the case of *AtCuAOζ* mutant, a reduced ROS level and stomatal closure in response to ABA are observed [25]. This evidence suggests a potential role *(AtCuAOβ*) or effective involvement *(AtCuAOζ*) in the control of the stomatal aperture in Arabidopsis [25,29]. Finally, and in agreement with our data, a CuAO was shown to act in *Vicia faba* during ABA responses involved in the regulation of stomatal apertures through the production of Put-derived H_2_O_2_ [22], suggesting the existence of a common hormonal response pathway in evolutionarily distant taxa.

The complexity of the ABA signal transduction in guard cells is also highlighted by evidence showing that multiple pathways involving several components and compartments are required in the control of stomatal movements in Arabidopsis. In this context, plasma membrane-located nicotinamide adenine dinucleotide phosphate (NADPH) oxidases (AtrbohD and F [53]), regulated by the ABA-induced phospholipase D (PLDα1 [25]), and ABA-activated OST1 [54], peroxisomal AtCuAOζ [25], and vacuolar AtCuAOδ (in this work) represent multiple ROS sources. These different ROS sources are active in different cellular compartments, targeted by ABA, and necessary for this response, suggesting that a strongly coordinated network is needed for the hormonal control of stomatal closure. Indeed, coordination of ROS signaling from different organelles has been reported for ABA-induced stomatal closure [55]. Furthermore, some evidence shows that ABA presents an apparently minor ROS-independent effect on stomatal closure, as its effects could not be entirely counter-balanced by either ROS scavengers or ROS-biosynthesis inhibitors (Figure 2, Table S1) [25].

Our data revealed that another player is necessary in ABA-mediated regulation of stomatal closure and points out the necessity of understanding the hierarchy of action or eventual synergy of actors involved. The contribution of both NADPH oxidases and AOs has also been proposed in the two phases of ROS production during the hypersensitive response to pathogens, with the former involved in the initial burst or first phase and the latter in the second phase [18,19]. It is possible that a similar mechanism involving both AtCuAOδ and AtCuAOζ, as well as NADPH oxidases, have a role in the ABA control of water balance homeostasis acting through cells or tissues responsible for the regulation of water loss in Arabidopsis. Of note is that an apoplastic PAO and a NADPH oxidase are involved in a feed-forward ROS amplification loop in tobacco, suggesting that both enzymes cooperate in ROS homeostasis in plants [37], while a peroxisomal PAO cross-talks with NADPH oxidase in Arabidopsis to activate mitochondrial alternative oxidase, underlining the complexity of ROS homeostasis and biosynthesis involving different enzymatic systems and subcellular compartments [56]. This picture is further enriched by the downstream effect of NO on the involvement of both NADPH oxidase and AOs in PA-induced stomatal closure in the guard cells of Arabidopsis [57]. The complexity of the events involved in the process of stomatal closure in cases where the increase of ABA levels are a signal of both immediate or prolonged reduced water potential could be explained on the basis of required differential and accurate responses at the cellular or tissue level to the different conditions to which plants are commonly exposed during their growth in-field. Clarification of these mechanisms would be helpful in elucidating potential applications for crop adaptation to changing climate, wherein water stress conditions are becoming increasingly relevant.

## 4. Materials and Methods 

### 4.1. Plant Materials, Growth Conditions and Treatments

The Columbia-0 (Col-0) ecotype of Arabidopsis was used as WT. The Arabidopsis Col-0 T-DNA insertion lines *Atcuaoδ.1* (SALK_072954.55.00.x line, TAIR accession number 4122972) and *Atcuaoδ.2* (GK-011C04-013046 line, TAIR accession number 4242275) of the gene (At4g12290 TAIR accession number 2139069) were obtained from the Salk Institute Genomic Analysis Laboratory (http://signal.salk.edu/tabout.html; [58]) and from the Bielefeld University CeBiTec/GABI-KAT III [59]. Information on the T-DNA insertion mutants *Atcuaoδ.1* and *Atcuaoδ.2* were obtained respectively from the SIGnAL website (http://signal.salk.edu) and from the GABI-KAT website (https://www.gabi-kat.de/). The Arabidopsis Col-0 transgenic lines constitutively expressing *AtCuAOδ* (*overAtCuAOδ*) were constructed as described below.

Plants were grown in soil or in vitro in a growth chamber at a temperature of 23 °C under long-day conditions (16/8 h photoperiod; 50 µmol m^−2^ s^−1^ and 55% relative humidity). Soil-grown plants were used for identification of the homozygous insertion mutants, for floral dip transformation to prepare over-expression lines, and in all cases for harvesting seeds of the WT and selected lines. For in vitro growth, seeds were surface sterilized as previously described [60]. Seeds were cold stratified at 4 °C and grown in one-half-strength Murashige and Skoog salt mixture supplemented with 0.5% (w/v) sucrose in presence of 0.8% (w/v) agar. In vitro-grown seedlings were used in the analysis of *AtCuAOδ* gene expression, in measurements of stomatal apertures, and in ROS detection.

The analysis of *AtCuAOδ* gene expression upon hormone treatment was performed on Arabidopsis seedlings grown for 12 days on agar medium and then transferred to liquid medium [one-half-strength Murashige and Skoog salt mixture supplemented with 0.5% (w/v) sucrose] containing 1, 10, and 100 μM abscisic acid (ABA; Duchefa) for the described time (0, 1, 3, 6, and 24 h).

Stomatal aperture measurements were performed on 12-day-old Arabidopsis WT plants, *Atcuaoδ* mutants, and *overAtCuAOδ* lines (homozygous T3 generation) grown on agar medium under control conditions in absence of treatment (Control WT, Control *Atcuaoδ.1*, Control *Atcuaoδ.2,* or *overAtCuAOδ* lines). For WT and mutants plants, stomatal aperture levels were also monitored after 2 h treatment with ABA (1, 10, and 100 μM), *N,N^1^*-dimethylthiourea (DMTU; 100 μM), 1, 10, and 100 μM ABA/100 μM DMTU, 2-bromoethylamine (2-BrEtA; 0.5, 5 mM), aminoguanidine (AG; 0.1, 1 mM), 100 μM ABA/0.5 or 5 mM 2-BrEtA, and 100 μM ABA/0.1 or 1 mM AG.

The detection of reactive oxygen species (ROS) in guard cells was analyzed on 12-day-old Arabidopsis WT plants, *Atcuaoδ* mutants, and *overAtCuAOδ* lines grown on agar medium under control conditions. ROS levels in WT and mutant plants were also analyzed after 2 h treatment with 100 μM ABA. 

### 4.2. Identification of the T-DNA Insertional Loss-of-Function Atcuaoδ.1 and Atcuaoδ.2 Mutants

Plants homozygous for the T-DNA insertion were identified by Polymerase Chain Reaction (PCR) on genomic DNA extracted from leaves of soil-grown plants by alkali treatment [61], using gene- and T-DNA-specific primers. *AtCuAOδ* gene-specific primers (*RP-AtCuAOδ*/*LP-AtCuAOδ*) were designed outside of the 5′ and 3′ ends of the T-DNA insertions and the T-DNA specific primers (*LBa1* for *Atcuaoδ.1*, and *RB1-pAC161* for *Atcuaoδ.2)* were designed at its left border (Figure S1 and Table S3). Due to the proximity of the insertion points in the two mutants, the same *AtCuAOδ* gene-specific primers were used for both the mutants and referred to as *RP-AtCuAOδ*/*LP-AtCuAOδ* (Figure S1). The genotype of the *Atcuaoδ* mutants was ascertained by two sets of PCR reactions: one using *RP-AtCuAOδ/LBa1* for *Atcuaoδ.1* and *LP-AtCuAOδ/RB1-pAC161* for *Atcuaoδ.2* to determine the presence of the T-DNA insertion and the other using *RP-AtCuAOδ*/*LP-AtCuAOδ* for both the mutants to verify the absence of the fragment indicative of a WT allele, as the T-DNA insertion originates a non-amplifiable long transcript (Figure S1). The absence of the full-length *AtCuAOδ* gene transcript in *Atcuaoδ.1* and *Atcuaoδ.2* seedlings was analyzed by Reverse Transcription Polymerase Chain Reaction (RT-PCR) of total RNA, using *rtPCR-AtCuAOδ-for1/RP-AtCuAOδ* as gene-specific primers (Figure S1 and Table S3), which would generate in WT an amplicon of 545 bp.

### 4.3. Construction of the Over-Expressing Transgenic Lines

The transgenic Arabidopsis *overAtCuAOδ* plants were prepared using Gateway technology. The *AtCuAOδ* gene sequence was amplified by PCR from *Arabidopsis* genomic DNA extracted by alkali treatment [61] from agar medium-grown seedlings using the gene-specific primers *overAtCuAOδ-for* and *overAtCuAOδ-rev* (Table S3). The *overAtCuAOδ-rev* primer was designed in order to insert the coding sequence for two Ser residues followed by a 6×His tag prior to the stop codon of the corresponding amplicon. The PCR product was purified and cloned initially into the pDONR 221 vector (Invitrogen), sequenced, and cloned into the pK2GW7 vector [62] through the Gateway recombination system (Invitrogen). The pK2GW7 construct *(35SCaMV::AtCuAOδ-6His)* was checked by sequencing prior to be transferred to *Agrobacterium tumefaciens* (strain GV 301) and then used to transform soil-grown *Arabidopsis* Col-0 WT plants by the floral dip transformation method [63]. Putatively transformed plants were controlled on selective medium (agar medium supplemented with kanamycin at the final concentration of 50 μg/mL) and subsequent PCR analysis of genomic DNA using the gene-specific primer *overAtCuAOδ-for* and a 6×His tag-specific primer (Table S3). Recombinant *AtCuAOδ* expression in *35SCaMV::AtCuAOδ-6His* transgenic plants was determined by RT-qPCR using the gene-specific primers *RTqPCR-AtCuAOδ-for* and the *RTqPCR-AtCuAOδ-rev* (Table S3), as well as western-blot analysis using a rabbit anti-6×His tag antibody conjugated to horseradish peroxidase (Abcam). All the analyses were performed on the third generation (T3) of the lines described herein.

### 4.4. PCR, RT-PCR and RT-Quantitative PCR (RT-qPCR) Analysis

PCR reactions was carried out with the Dream*Taq*TM DNA Polymerase (Fermentas) in a iCyclerTM ThermalCycler (Bio-Rad) with the following parameters: 2 min of denaturation at 95 °C, 35 cycles of 95 °C for 30 s, 58 °C for 1 min, 72 °C for 1.5 min, and 10 min at 72 °C for the final extension. Total RNA was isolated from 12-day-old whole Arabidopsis seedlings using the RNeasy Plant Mini kit (QIAGEN) following the manufacturer’s instructions. DNase digestion was performed during RNA purification using the RNase-Free DNase Set (QIAGEN). For RT-PCR, the first cDNA strand was synthesized from total RNA following the protocol of the ImProm-II Reverse Transcription System (Promega). Ubiquitin-conjugating enzyme 21 (*UBC21)* [64] was used as the internal control to confirm equal amounts of RNA among the various samples, using the primers *UBC21-for* and *UBC21-rev* (Table S3).

RT-qPCR analysis was performed on DNase-treated RNA (4 μg) from 12-day-old whole Arabidopsis seedlings. The cDNA synthesis and PCR amplification were carried out using GoTaq® 2-Step RT-qPCR System200 (Promega) according to the manufacturer’s protocol. The PCRs were run in a Corbett RG6000 (Corbett Life Science, QIAGEN) utilizing the following program: 95 °C for 2 min, then 40 cycles of 95 °C for 7 s and 60 °C for 40 s. The melting program ramps from 60 °C to 95 °C. rising by 1 °C each step. *AtCuAOδ* specific primers were *RTqPCR-AtCuAOδ-for* and *RTqPCR- AtCuAOδ-rev* (Table S3). *UBC21* (At5g25760) was used as reference gene and specific primers were prepared [64] (*UBC21-For* and *UBC21-Rev*; Table S3). Fold change in the expression of the *AtCuAOδ* was calculated according to the ΔΔC_q_ method as follows [65], where:

C_q_ refers to the quantification cycle,
ΔC_q_ = C_q target-gene_ − C_q reference-gene_,
ΔΔC_q_ = 2^–[ΔC^_q sample time X_^– ΔC^_q control-sample time 0_^]^,
Expression fold-induction = ΔΔC_q treated sample_ / ΔΔC_q non-treated sample_.

The value of ΔC_q_ at time 0 (control sample) has been assumed to be the reference value for both treated and untreated samples at each experimental time. Accordingly, ΔΔC_q_ has been calculated as indicated above for both treated and untreated samples at each experimental time. The reported values of expression fold-inductions after treatment are relative to the corresponding expression values of non-treated plants for each time point, with the value for time zero assumed to be one. The software used to control the thermocycler and to analyze data was the Corbett Rotor-Gene 6000 Application Software (version 1.7, Build 87; Corbett Life Science, QIAGEN, Milan, Italy).

### 4.5. Measurement of Stomatal Aperture

Measurement of stomatal aperture was performed as described previously [66], with slight modifications. In detail, seedlings from 12-day-old Arabidopsis WT plants, *Atcuaoδ* mutants, and *AtCuAOδ* over-expressing lines grown on agar medium were incubated in opening solution (30 mM KCl, 10 mM MES-Tris, pH 6.15) for 3 h under light to allow stomatal opening. Then, seedlings from WT plants and *Atcuaoδ* mutants were incubated for 2 h under light in liquid medium in the absence or presence of ABA 1, 10, and 100 µM, DMTU 100 µM, and ABA 1, 10, and 100 μM/DMTU 100 μM to analyze stomatal aperture.

Treatments with CuAOs inhibitors 2-BrEtA and AG were performed as follows: after 3 h incubation with opening solution, seedlings from WT plants and *Atcuaoδ* mutants were incubated in liquid medium supplemented or not with 2-BrEtA (0.5, 5 mM) or AG (0.1, 1 mM) for 30 min under light, after which ABA at the final concentration of 100 μM was added and further incubated for 2 h under light.

Based on preliminary experiments of ABA dose-response curve, independent experiments were carried out grouping treatments in separate blocks by ABA concentration or CuAO inhibitor type.

Following the various treatments, seedlings from WT plants, *Atcuaoδ* mutants, and *AtCuAOδ* over-expressing lines were treated with a fixing solution (1% glutaraldehyde, 10 mM NaPi pH 7.0, 5 mM MgCl_2_, and 5 mM EDTA) and incubated for 30 min under light. Images of stomata with the outline of stomatal pores in the focal plane were acquired by a Leica DFC 450C digital camera applied to a Zeiss Axiophot 2 microscope at the magnification of 20×, and stomatal apertures (width/length) were measured using a digital ruler (ImageJ 1.44). Width and length of stomata pores were measured, and stomatal apertures were expressed as the width/length ratio.

### 4.6. In Situ Detection of Reactive Oxygen Species (ROS) in Guard Cells

ROS production in guard cells was analyzed using a chloromethyl derivative of 2′,7′-dichlorodihydrofluorescein diacetate (CM-H_2_DCFDA; Molecular Probes, Invitrogen) as previously described [25,67], with slight modifications. Arabidopsis leaves from 12-day-old seedlings from WT, as well as *AtCuAOδ* insertional mutants and over-expressing lines grown on agar medium, were detached and incubated for 3 h in the assay solution containing 5 mM KCl, 50 μM CaCl_2_, and 10 mM MES-Tris (pH 6.15), and then 50 μM CM-H_2_DCFDA was added to the sample. Leaves were incubated for 30 min at room temperature and then the excess dye was washed out with the fresh assay solution. Collected tissues were again incubated in the assay solution containing 100 μM ABA for 20 min in dark conditions. Images were captured by Laser Scanning Confocal Microscopy (LSCM), using a Leica TCS-SP5 equipped with an Argon laser (Excitation/Emission: ~492–495/517–527 nm) and the Leica Application Suite Advanced Fluorescence (LAS-AF; Leica Microsystems, Milan, Italy).

### 4.7. Statistics

For RT-qPCR analysis, five independent experiments (in this case representing the biological replicates; *n* = 5) were performed; in each experiment, the seedlings (~200 mg) from four agar plates for every time point and treatment were used. For each cDNA obtained, qPCR was performed in triplicate (technical replicates) and the triplicate mean values have been used in the statistical analysis for each of the five independent experiments.

For the stomatal aperture measurements, three independent experiments were performed for each treatment on the different genotypes. Independent experiments were carried out grouping treatments in separate blocks by ABA concentration or CuAO inhibitor type. A Control and a 100 µM ABA treatment for each of the genotypes analyzed were always included to verify data reproducibility between blocks. For each time, five similarly-sized leaves were harvested from different seedlings for each genotype and treatment. In this case, each of the five leaves from the three experiments was considered a biological replicate for a total of fifteen biological replicates for each genotype and treatment (*n* = 15). For each leaf, four random chosen fields (430 µm × 325 µm) were acquired and approximately 60 stomata were measured, and the mean values were used in the statistical analysis. The results presented are supported by preliminary experiments of ABA dose-response curves. Statistical tests were performed using GraphPad Prism (GraphPad Software) with one-way ANOVA analysis, followed by Sidak’s multiple comparison tests. Statistical significance of differences was evaluated by *p* level, with ns showing not significant, and *, **, ***, and **** *p* values representing equal or less than 0.05, 0.01, 0.001, and 0.0001, respectively. The LSCM analysis of ROS production by CM-H_2_DCFDA-staining was performed on plants from five independent experiments, each of which analyzed leaves from five plants per plant genotype and per treatment, yielding reproducible results. Images from single representative experiments are shown.

## Supplementary Materials

The following are available online at https://www.mdpi.com/2223-7747/8/6/183/s1. Figure S1: Characterization of *Atcuaoδ.1* and *δ.2* mutants. Figure S2: Nucleotide and deduced amino acid sequences of *AtCuAOδ* gene (At4g12290, TAIR accession number 2139069) retrieved from TAIR database. Figure S3: Characterization of the lines over-expressing the *AtCuAOδ* gene, showing both protein and mRNA levels and the positive correlation between them. Table S1: Data presented in Figure 2A with the effect of ABA and DMTU on stomata pore width/length ratio. Results from three independent experiments are reported (mean values, SD, and SE). Table S2: Data presented in Figure 2B with the effect of ABA and CuAO inhibitors (2-BrEtA and AG) on stomata pore width/length ratio. Results from three independent experiments are reported (mean values, SD, and SE). Table S3: Primers used in the different PCR procedures indicated in Material and Methods.
