# Supplementary material for: The Copper Amine Oxidase AtCuAOδ Participates in Abscisic Acid-Induced Stomatal Closure in Arabidopsis
Source: Plants (Basel). 2019 Jun 20;8(6):183. doi: 10.3390/plants8060183 (PMC6630932; doi:10.3390/plants8060183)
Supplement: Supplementary file 1 [file plants-08-00183-s001.pdf]

Supplementary Information

Supplemental Table S1. Data presented in Figure 2A with the effect of ABA and DMTU on stomatal pore width/length ratio of 12-day-old seedlings from WT, *Atcuaoδ.1* and *Atcuaoδ.2.* Seedlings were treated for 2 h with ABA (1, 10, 100 µM) and DMTU (100 µM) either alone or in combination with the hormone. Mean values, SD and SE (*n* = 15) are reported.

**WT**								
	**Control**	**ABA 1 µM**	**ABA 10 µM**	**ABA 100 µM**	**DMTU 100 µM**	**ABA 1 µM + DMTU**	**ABA 10 µM + DMTU**	**ABA 100 µM + DMTU**
**Mean**	0,703	0,346	0,286	0,166	0,733	0,640	0,560	0,549
**SD**	0,122	0,101	0,063	0,063	0,112	0,139	0,109	0,115
**SE**	0,012	0,012	0,007	0,006	0,012	0,022	0,015	0,021
Atcuaoδ.1								
	**Control**	**ABA 1 µM**	**ABA 10 µM**	**ABA 100 µM**	**DMTU 100 µM**	**ABA 1 µM + DMTU**	**ABA 10 µM + DMTU**	**ABA 100 µM + DMTU**
**Mean**	0,708	0,646	0,622	0,603	0,735	0,691	0,660	0,651
**SD**	0,118	0,115	0,125	0,098	0,099	0,091	0,139	0,104
**SE**	0,011	0,016	0,017	0,009	0,012	0,015	0,027	0,011
Atcuaoδ.2								
	**Control**	**ABA 1 µM**	**ABA 10 µM**	**ABA 100 µM**	**DMTU 100 µM**	**ABA 1 µM + DMTU**	**ABA 10 µM + DMTU**	**ABA 100 µM + DMTU**
**Mean**	0,707	0,621	0,609	0,589	0,737	0,658	0,646	0,632
**SD**	0,122	0,133	0,104	0,090	0,112	0,058	0,110	0,112
**SE**	0,011	0,017	0,014	0,008	0,012	0,012	0,022	0,012

Supplemental Table S2. Data presented in Figure 2B with the effect of ABA and CuAO inhibitors (2-BrEtA and AG) on stomatal pore width/length ratio of 12-day-old seedlings from WT, *Atcuaoδ.1* and *Atcuaoδ.2.* Seedlings were treated with 2-BrEtA (0.5, 5 mM) or AG (0.1, 1 mM) for 30 min. ABA was added (100 µM) and further incubated for 2 h.Mean values, SD and SE (*n* = 15) are reported.

WT										
	**Control**	**ABA 100 µM**	**2-BrEtA 0,5 mM**	**ABA + 2-BrEtA 0,5 mM**	**2-BrEtA 5 mM**	**ABA + 2-BrEtA 5 mM**	**AG 0,1 mM**	**ABA + AG 0,1 mM**	**AG 1 mM**	**ABA + AG 1 mM**
**Mean**	0,702	0,166	0,734	0,523	0,734	0,630	0,634	0,455	0,635	0,536
**SD**	0,116	0,050	0,099	0,078	0,124	0,103	0,101	0,079	0,095	0,085
**SE**	0,016	0,005	0,014	0,010	0,015	0,014	0,014	0,010	0,012	0,011

Atcuaoδ.1										
	**Control**	**ABA 100 µM**	**2-BrEtA 0,5 mM**	**ABA + 2-BrEtA 0,5 mM**	**2-BrEtA 5 mM**	**ABA + 2-BrEtA 5 mM**	**AG 0,1 mM**	**ABA+ AG 0,1 mM**	**AG 1 mM**	**ABA + AG 1 mM**
**Mean**	0,708	0,605	0,704	0,638	0,712	0,665	0,635	0,599	0,637	0,597
**SD**	0,124	0,090	0,118	0,095	0,121	0,101	0,094	0,074	0,117	0,089
**SE**	0,013	0,010	0,015	0,013	0,015	0,014	0,013	0,010	0,016	0,012

Atcuaoδ.2										
	**Control**	**ABA 100 µM**	**2-BrEtA 0,5 mM**	**ABA + 2-BrEtA 0,5 mM**	**2-BrEtA 5 mM**	**ABA + 2-BrEtA 5 mM**	**AG 0,1 mM**	**ABA+ AG 0,1 mM**	**AG 1 mM**	**ABA + AG 1 mM**
**Mean**	0,703	0,589	0,646	0,590	0,659	0,637	0,626	0,583	0,638	0,585
**SD**	0,129	0,092	0,121	0,105	0,126	0,105	0,107	0,085	0,105	0,077
**SE**	0,016	0,010	0,016	0,013	0,016	0,014	0,014	0,012	0,013	0,010

Supplemental Table S3. Primers used in the different PCR procedures indicated in Material and Methods.

**Reactions**	**Primer name**	**Primer sequence**
T-DNA insertion analysis left border specific for *Atcuaoδ.1*	*LBa1*	5′- GATGGTTCACGTAGTGGGCCATCGC-3′
T-DNA insertion analysis left border specific for *Atcuaoδ.2*	*RB1-pAC161*	5’- GAAGATAGTGGAAAAGGAAGGTGGCTC-3’
T-DNA insertion analysis gene specific 1; forward	*LP-AtCuAOδ*	5′-GTCCTCCATTTTTCAATCGTC-3′
T-DNA insertion analysis gene specific 2; reverse	*RP-AtCuAOδ*	5′-TCGGTTATCTCGATCACTTGC-3′
Genomic amplification for Gateway cloning; forward	*overAtCuAOδ-for*	5'-GGGGACAAGTTTGTACAAAAAAGCAGGCT ATGGACCAAAAAAGCTTTTTCCGG-3'
Genomic amplification for Gateway cloning; reverse	*overAtCuAOδ-rev*	5’-GGGGACCACTTTGTACAAGAAAGCTGGGT TCAGTGGTGGTGGTGGTGGTGTCCTCCAGCA GAAACAGATTGAACTCCACAAACC-3'
Over-expression line analysis His tag; reverse	6×His tag-specific primer	5’-GGGGACCACTTTGTACAAGAAAGCTGGGT CTAGTGGTGGTGGTGGTGGTGTCCTC-3’
Gene specific primer for RT-PCR; forward	*rtPCR-AtCuAOδ-for1*	5’-GACCCACTCACGGTGTCGGAG-3’
Gene specific primer for RT-qPCR; forward	*RTqPCR-AtCuAOδ-for*	5′- GATGACACTCTTGCAGTTTGG -3′
Gene specific primer for RT-qPCR; reverse	*RTqPCR-AtCuAOδ-rev*	5′- GGAATGTGATGGAAACCAAGTG -3′
Control *UBC21* gene specific primer for RT-PCR; forward	*UBC21-for*	5′- CTGCGACTCAGGGAATCTTCTAA -3′
Control *UBC21* gene specific primer for RT-PCR; reverse	*UBC21-rev*	5′- TTGTGCCATTGAATTGAACCC -3′

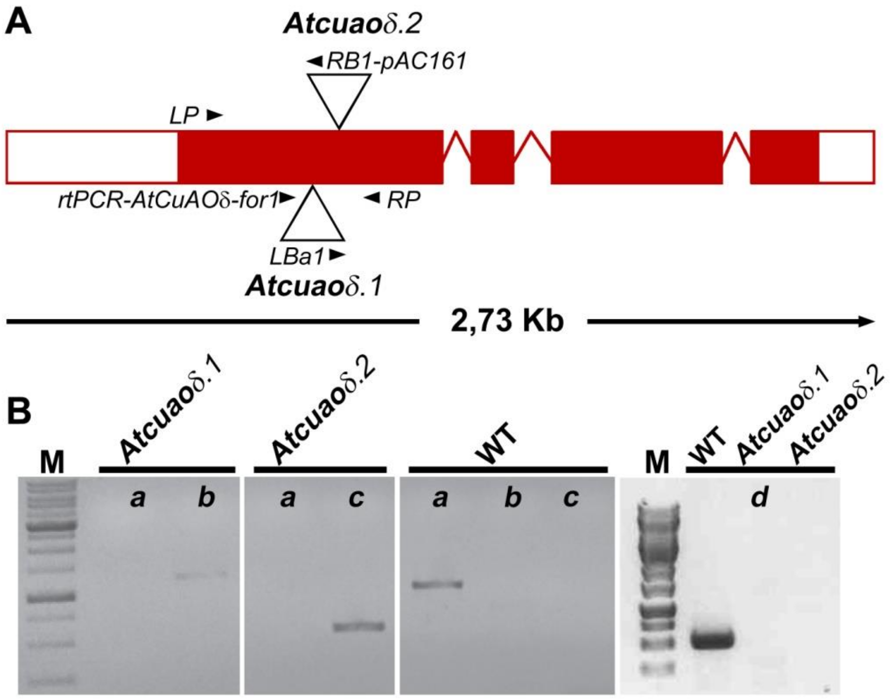

Supplemental Figure S1. Characterization of *Atcuaoδ.1* and *δ.2* mutants. (**A**) T-DNA insertion positions responsible for the two mutations are indicated in the genomic region encoding the copper-amino-oxidase, *AtCuAOδ* (At4g12290, TAIR accession number 2139069). The position of the primers used to characterize the insertions and the potential transcription of a truncated mRNA, are also represented. (**B)** Gel analysis of PCR and Rt-PCR products from genomic DNA or cDNA from WT and Arabidopsis mutants with the indicated set of primers (set **a** to **d**) where the predicted amplicons were observed as follows: set **a.** *AtCuAOδ* gene specific primers: *LP-Atcuao*δ*/RP-Atcuao*δ with a predicted amplicon of 1100bp from WT genomic DNA; set **b.** gene specific and SALK_072954.55.00.x insertion line (TAIR accession number 4122972): *RP-Atcuao*δ*/LBa1*; with predicted amplicon of 650bp from *Atcuaoδ.1* genomic DNA; set **c.** gene specific and GK-011C04-013046 insertion line (TAIR accession number 4242275): *LP-Atcuao*δ*/RB1-pAC161*; with predicted amplicon of 1400 bp from *Atcuaoδ.2* genomic DNA; set **d.** *AtCuAOδ* gene specific primers used for Rt-PCR: *rtPCR-AtCuAOδ-for1*/*RP-Atcuao*δ; with a predicted amplicon of 545 bp from Arabidopsis WT or mutant cDNA.

AtCuAOδ (At4g12290) T-DNA insertion mutants

```
ATG GAC CAA AAA AGC TTT TTC CGG TTG ATT TTT CTC ATA GTC ACA GCT GGT TTC ATC ATC TCC TTC ACT TCC ACT AAC TTC CCT CAC GCG
M   D   Q   K   S   F   F   R   L   I   F   L   I   V   T   A   G   F   I   I   S   F   T   S   T   N   F   P   H   A
CCG ACG AGG CTT CTT GAT TGC ACC GAC TCT TCT TCT TCA CCT CTT TGC GCT TCG AGA AAC TTT CTT TTC AAC AAA CAA CAA CCA CGA CCT
P   T   R   L   L   D   C   T   D   S   S   S   S   P   L   C   A   S   R   N   F   L   F   N   K   Q   Q   P   R   P
ATT CCT AAA CAT GAT CCT AAA CCA AAT ACC AAA AAC CAT GAT CAC GTG TCC GAT ACA CCA AAC CAT CCT CTA GAC CCA CTC ACG GTG TCG
I   P   K   H   D   P   K   P   N   T   K   N   H   D   H   V   S   D   T   P   N   H   P   L   D   P   L   T   V   S
GAG ATT AAC AAA ATC CGA TCA ATA CTC TCT TCT CAC GCG CTA TTC ACT TCC GGT ACA CCA CAC GCG CTC CAC ACC GTC GTT CTT GAA GAG
E   I   N   K   I   R   S   I   L   S   S   H   A   L   F   T   S   G   T   P   H   A   L   H   T   V   V   L   E   E
CCT GAG AAG AAT CTT GTC CGT CAC TGG GAA AAA GGG AAC CCA CTT CCT CCG AGG AAA GCT TCC GTC ATC GCA CGT GTT GGC GCG GAC ACG
P   E   K   N   L   V   R   H   W   E   K   G   N   P   L   P   P   R   K   A   S   V   I   A   R   V   G   A   D   T
CAC GTG CTC ACC GTT GAT ATC TCT ACG GGT CGG GTA GAT TCA GAG AAT AGC CCG GTT CGT GTT TCT GGT TAC CCG ATG ATG ACT ATA GAA
H   V   L   T   V   D   I   S   T   G   R   V   D   S   E   N   S   P   V   R   V   S   G   Y   P   M   M   T   I   E
```

Atcuaoδ.1

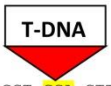


```
GAG ATG AAC GAT ATC ACT GTT GTA CCA TTT TCA AAC GCG GAT TTC AAC CGT ACG ATC ATC TCT CGT GGA GTT AAT CTA ACG GAC GTG ATT
E   M   N   D   I   T   V   V   P   F   S   N   A   D   F   N   R   T   I   I   S   R   G   V   N   L   T   D   V   I
```

Atcuaoδ.2

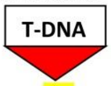


```
TGT TTC CCA ATC TCT TGT GGT TGG TTT GGT AAT AAA GAA GAA AAC GCG AGG GTA ATT AAA AGT CAG TGT TTC ATG ACA CAA GGA ACA CCT
C   F   P   I   S   C   G   W   F   G   N   K   E   E   N   A   R   V   I   K   S   Q   C   F   M   T   Q   G   T   P
AAC TTC TAC ATG CGT CCT ATC GAA GGT TTA ACC ATT CTC ATC GAT TTA GAT ACA AAG CAA GTG ATC GAG ATA ACC GAT ACA GGT CGG GCT
N   F   Y   M   R   P   I   E   G   L   T   I   L   I   D   L   D   T   K   Q   V   I   E   I   T   D   T   G   R   A
ATA CCC ATA CCC GGT TCA ACC AAT ACC GAT TAC CGC TTC CAA AAG CTC GCA ACC ACC GAC AAA ACT CGG CCT CTA AAC CCG ATA TCC ATT
I   P   I   P   G   S   T   N   T   D   Y   R   F   Q   K   L   A   T   T   D   K   T   R   P   L   N   P   I   S   I
GAG CAG CCA CGT GGT CCA AGC TTC GTG ATA GAG GAC AAC CAT CTA GTG AAA TGG GCA AAT TGG GAA TTT CAT CTA AAA CCT GAC CCG AGA
E   Q   P   R   G   P   S   F   V   I   E   D   N   H   L   V   K   W   A   N   W   E   F   H   L   K   P   D   P   R
GCA GGT GTG GTA ATA TCA CGG GTA AGA GTA CAC GAC CCG GAT ACT CAT GAG ACA CGT GAC GTG ATG TAC AAA GGT TTC GTG TCT GAA CTT
A   G   V   V   I   S   R   V   R   V   H   D   P   D   T   H   E   T   R   D   V   M   Y   K   G   F   V   S   E   L
TTT GTT CCG TAC ATG GAT CCA TCG GAC GCG TGG TAC TTT AAG ACT TAC ATG GAC GCA GGG GAA TAC GGG TTC GGG TTA CAA GCC ATG CCA
F   V   P   Y   M   D   P   S   D   A   W   Y   F   K   T   Y   M   D   A   G   E   Y   G   F   G   L   Q   A   M   P
CTC GTA CCG CTT AAT GAT TGT CCA CGA AAC GCA GCC TAT ATG GAC GGA GTT TTC GCC GCA GCC GAT GGA ACA CCG TTC GTG AGA GAA AAC
L   V   P   L   N   D   C   P   R   N   A   A   Y   M   D   G   V   F   A   A   A   D   G   T   P   F   V   R   E   N
ATG GTT TGT ATC TTT GAG AGT TAC GCC GGA GAT ATT GGG TGG CGT CAC TCC GAA AGC CCC ATC ACC GGT ATA CCG ATA AGG GAA GTG AGA
M   V   C   I   F   E   S   Y   A   G   D   I   G   W   R   H   S   E   S   P   I   T   G   I   P   I   R   E   V   R
CCA AAA GTG ACG CTA GTG GTA CGA ATG GCA GCT TCG GTA GGT AAC TAT GAT TAC ATC ATT GAT TAC GAG TTC CAA ACT GAT GGG CTT ATA
P   K   V   T   L   V   V   R   M   A   A   S   V   G   N   Y   D   Y   I   I   D   Y   E   F   Q   T   D   G   L   I
AAA GCT AAG GTC GGG CTA AGT GGA ATA CTA ATG GTG AAA GGG ACA ACA TAT CAA AAC AAA AAC CAA GTG GAG AAA GAT AAA GAC GGT AAT
K   A   K   V   G   L   S   G   I   L   M   V   K   G   T   T   Y   Q   N   K   N   Q   V   E   K   D   K   D   G   N
GAA GAA GAG CTT CAC GGC ACG CTT CTG TCT GAA AAT GTA ATT GGA GTA ATA CAC GAT CAC TAC GTC ACT TTT TAC CTT GAC CTT GAC GTC
E   E   E   L   H   G   T   L   L   S   E   N   V   I   G   V   I   H   D   H   Y   V   T   F   Y   L   D   L   D   V
GAT GGC CCG GAC AAC TCA TTT GTT AAA GTG AAT CTC AAG AGG CAA GAG ACC GAG CCA GGC GAG TCA CCG AGG AAA AGT TAC CTA AAA GCT
D   G   P   D   N   S   F   V   K   V   N   L   K   R   Q   E   T   E   P   G   E   S   P   R   K   S   Y   L   K   A
GTT AGG AAC ATT GCG AAA ACC GAA AAG GAT GGT CAG ATC AAG CTT AGC TTG TAC GAT CCA TCA GAA TTC CAC GTC ATC AAC TCT GGT AAA
V   R   N   I   A   K   T   E   K   D   G   Q   I   K   L   S   L   Y   D   P   S   E   F   H   V   I   N   S   G   K
ACC ACT CGG GTC GGA AAC CCG ACG GGT TAT AAG GTC GTT CCT AGA ACG ACG GCA GCT AGT CTA CTT GAC CAT GAT GAT CCG CCG CAG AAG
T   T   R   V   G   N   P   T   G   Y   K   V   V   P   R   T   T   A   A   S   L   L   D   H   D   D   P   P   Q   K
AGA GGA GCT TTT ACC AAC AAC CAA ATT TGG GTC ACT CCG TAC AAT AAG TCG GAG CAA TGG GCT GGT GGC TTG TTC ACT TAC CAA AGC CAT
R   G   A   F   T   N   N   Q   I   W   V   T   P   Y   N   K   S   E   Q   W   A   G   G   L   F   T   Y   Q   S   H
GGT GAT GAC ACT CTT GCA GTT TGG TCA GAC AGG GAT AGA GAC ATA GAG AAC AAG GAT ATA GTT GTG TGG TAT ACA CTT GGT TTC CAT CAC
G   D   D   T   L   A   V   W   S   D   R   D   R   D   I   E   N   K   D   I   V   V   W   Y   T   L   G   F   H   H
ATT CCA TGT CAA GAA GAT TTT CCG ATA ATG CCC ACG GTT TCT TCG AGT TTC GAT TTG AAG CCC GTA AAC TTT TTC GAG CGC AAT CCA ATC
I   P   C   Q   E   D   F   P   I   M   P   T   V   S   S   S   F   D   L   K   P   V   N   F   F   E   R   N   P   I
CTC AGT GCC GCT CCA AAC TTT GAA CAT GAT CTC CCG GTT TGT GGA GTT CAA TCT GTT TCT GCT TGA
L   S   A   A   P   N   F   E   H   D   L   P   V   C   G   V   Q   S   V   S   A   STOP
```

Supplemental Figure S2. Nucleotide and deduced amino acid sequences of *AtCuAOδ* gene (At4g12290, TAIR accession number 2139069) retrieved from TAIR database. The red triangles represent the sites of T-DNA insertion in *Atcuaoδ.1* (SALK_072954.55.00.x line, TAIR accession number 4122972) and *Atcuaoδ.2* (GK-011C04-013046 line, TAIR accession number 4242275) mutants according to TAIR database. The five active site residues essential for catalysis (Asp300, Tyr387, His442, His444 and His603; *Pisum sativum* CuAO numbering) are highlighted respectively in green, red (Tyr residue precursor of the TPQ cofactor) and in yellow (the three His involved in copper coordination).

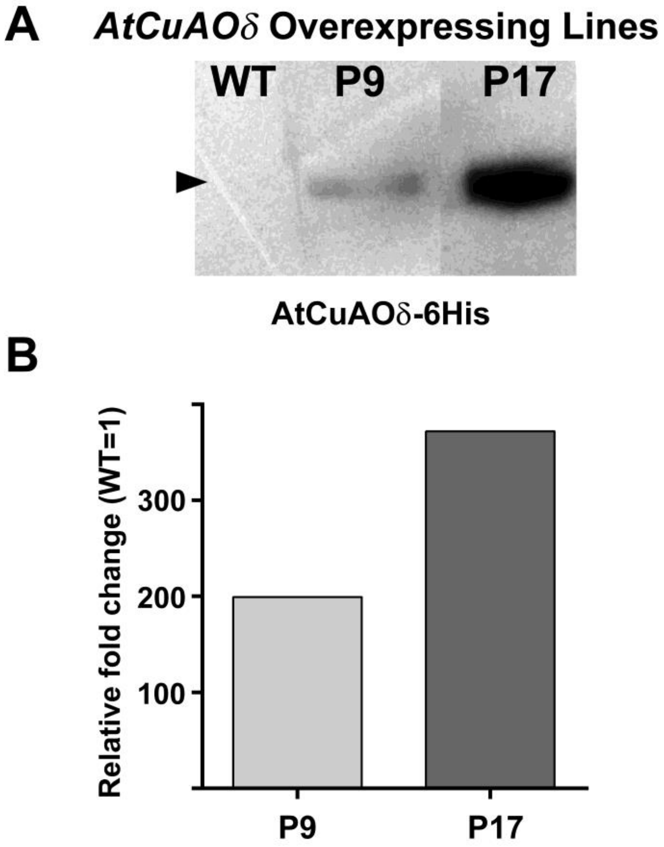

Supplemental Figure S3. Characterization of the lines over-expressing the *AtCuAOδ* gene, showing both protein and mRNA levels and the positive correlation between them.. (**A)** Western blot analysis of crude extracts from WT plants and two *overAtCuAOδ* lines using an anti-His tag antibody, after SDS-PAGE, loaded on the basis of total protein content. (**B)** Quantitative RT-PCR of *AtCuAOδ* in the over-expressing lines showing the level of mRNA as compared to WT (WT=1).
